# Temporal stability of intracranial electroencephalographic abnormality maps for localizing epileptogenic tissue

**DOI:** 10.1111/epi.17663

**Published:** 2023-06-06

**Authors:** Yujiang Wang, Gabrielle M. Schroeder, Jonathan J. Horsley, Mariella Panagiotopoulou, Fahmida A. Chowdhury, Beate Diehl, John S. Duncan, Andrew W. McEvoy, Anna Miserocchi, Jane de Tisi, Peter N. Taylor

**Affiliations:** ^1^ CNNP Lab, Interdisciplinary Computing and Complex BioSystems Group, School of Computing Newcastle University Newcastle Upon Tyne UK; ^2^ Faculty of Medical Sciences Newcastle University Newcastle Upon Tyne UK; ^3^ UCL Queen Square Institute of Neurology Queen Square London UK

## Abstract

**Objective:**

Identifying abnormalities on interictal intracranial electroencephalogram (iEEG), by comparing patient data to a normative map, has shown promise for the localization of epileptogenic tissue and prediction of outcome. The approach typically uses short interictal segments of approximately 1 min. However, the temporal stability of findings has not been established.

**Methods:**

Here, we generated a normative map of iEEG in nonpathological brain tissue from 249 patients. We computed regional band power abnormalities in a separate cohort of 39 patients for the duration of their monitoring period (.92–8.62 days of iEEG data, mean = 4.58 days per patient, >4800 hours recording). To assess the localizing value of band power abnormality, we computed $D_{\mathrm{RS}}$—a measure of how different the surgically resected and spared tissue was in terms of band power abnormalities—over time.

**Results:**

In each patient, the $D_{\mathrm{RS}}$ value was relatively consistent over time. The median $D_{\mathrm{RS}}$ of the entire recording period separated seizure‐free (International League Against Epilepsy [ILAE] = 1) and not‐seizure‐free (ILAE > 1) patients well (area under the curve [AUC] = .69). This effect was similar interictally (AUC = .69) and peri‐ictally (AUC = .71).

**Significance:**

Our results suggest that band power abnormality D_RS, as a predictor of outcomes from epilepsy surgery, is a relatively robust metric over time. These findings add further support for abnormality mapping of neurophysiology data during presurgical evaluation.

**Key Points**

- Normative maps measure expected healthy variations in iEEG band power
- Band power abnormalities hold localizing value for identifying the epileptogenic zone and predicting outcome
- We investigated the temporal stability of band power abnormalities in almost 5000 hours of recordings in 39 patients
- Predictions of outcome, using the abnormalities, were relatively stable over time; median predictions discriminated groups with AUC = .69
- These findings pave the way to clinical translation of abnormality mapping during presurgical evaluation

## 1 INTRODUCTION

Interictal electroencephalographic (EEG) biomarkers are currently under active research to help localize the epileptogenic zone (EZ), but a key question is their temporal stability. Increasing evidence suggests that some markers, such as EEG spikes^1^ and high‐frequency oscillations,^2^ are not static, with some studies concluding that long multiday recordings are required to observe the full range of variability in the interictal markers. To date, there has not been a direct investigation of the temporal impact on localization ability validated with surgical outcome.

Normative band power maps have recently emerged as a promising approach to identify the EZ, and we will focus on this approach as our interictal biomarker in this study. In a given patient, the approach can be summarized as *z*‐scoring interictally observed band power in a particular brain region to a normative control distribution of expected data in said region, thus deriving a regional band power abnormality. With intracranial EEG (iEEG), obtaining a normative distribution is particularly challenging, as healthy control data are not available. Instead, the normative distribution is derived from recordings from postulated nonepileptic brain areas across a large cohort of patients.^3^, ^4^, ^5^ The subsequently derived band power abnormalities have recently been shown by multiple independent studies to contain localizing information.^6^, ^7^, ^8^ However, those studies used short segments of EEG or magnetoencephalographic (MEG) recordings of approximately 1 min far away from seizures. It is unclear how the localizing ability of the approach might fluctuate over time.

EEG—specifically, iEEG band power—has been repeatedly shown to fluctuate over a range of timescales, and specifically circadian fluctuations have been consistently reported.^9^, ^10^, ^11^, ^12^, ^13^ Previous work also highlights that EEG features may change peri‐ictally.^14^, ^15^ It is therefore reasonable to assume that band power scored against a static normative map also fluctuates over time. Thus, an important next step is to investigate how band power abnormalities change over time.

In this work, we investigate band power abnormalities over time in sessions of iEEG monitoring for presurgical epilepsy diagnostics, which typically span multiple days. We evaluate how temporal changes affect our ability to localize epileptogenic tissue by investigating how well we can distinguish tissue that was later resected versus spared based on band power abnormality in each brain region over time. We further test whether there are specific peri‐ictal changes in our ability to distinguish resected and spared tissue and validate all results with patient outcomes of postsurgical seizure freedom.

## 2 MATERIALS AND METHODS

**FIGURE 1:**
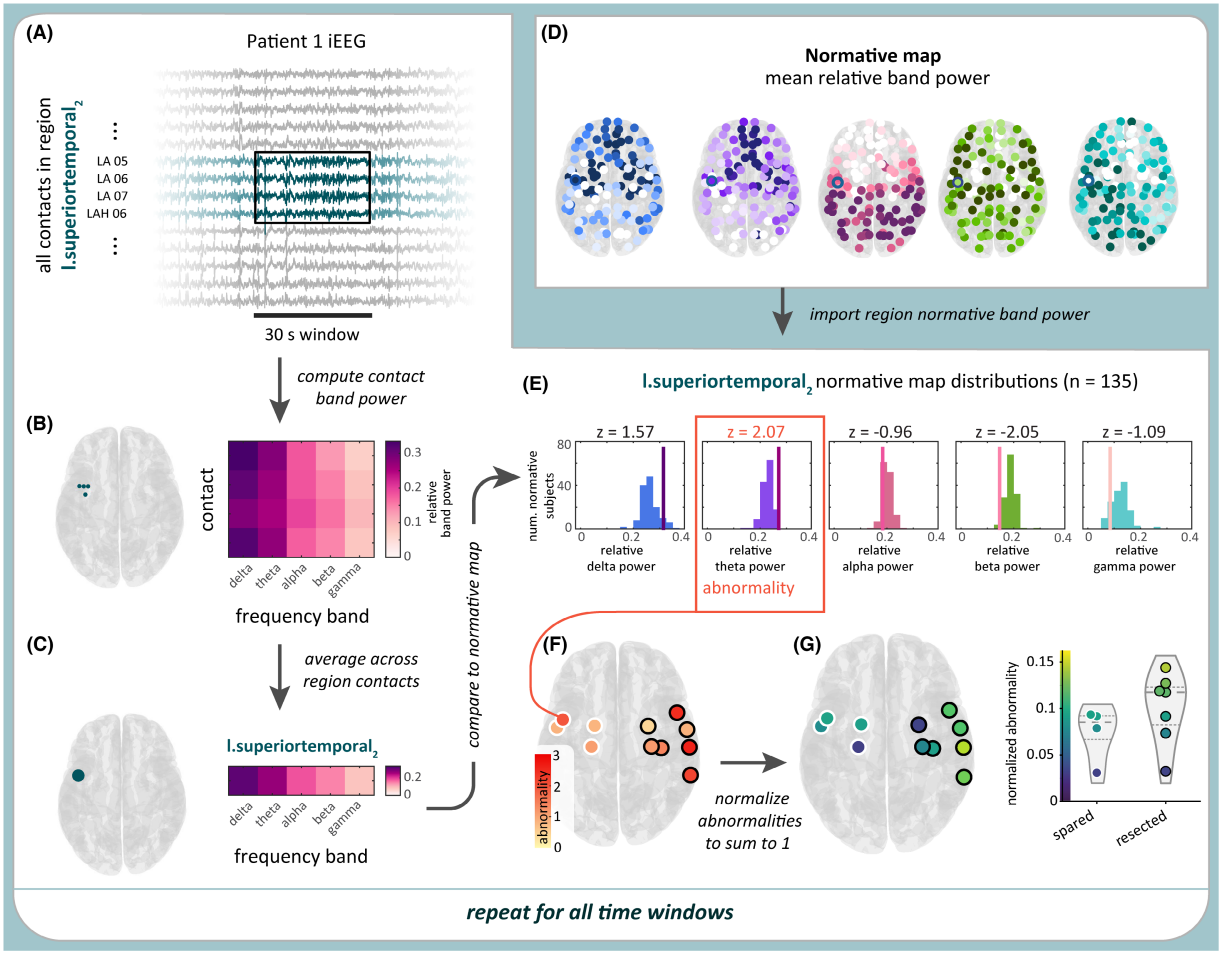
Computing band power abnormality in a sample time window and region in Patient 1. (A) Sample 30‐s time window of intracranial electroencephalographic (iEEG) data in a subset of Patient 1's contacts. All contacts within a sample region, l.superiortemporal_2_, are highlighted in blue. (B) From the 30 s of iEEG data, the relative log band power of each of the four contacts in the sample region was computed. (C) Averaging relative log band power across all of the region's contacts produces the region's relative band power. (D) Relative log band power was also computed in a separate cohort of 249 subjects, yielding a normative map of this measure. (E) Patient 1's regional relative band power was then *z*‐scored relative to the normative map. The region's abnormality was defined as the maximum absolute *z*‐score (here, 2.07) across the five frequency bands. (F) The process is repeated for all regions. (G) The abnormality values are normalized so their sum equals 1 and plotted for resected and spared regions. This process was repeated for all time windows in each patient.

Our approach was to compute a normative map of interictal iEEG band power to which patients from an independent site can be compared. Then, in comparing patients to the normative map, we could compute band power abnormality, with the expectation that if abnormalities were present beyond the resection, the patient will not be seizure‐free. We used preoperative magnetic resonance imaging (MRI), postimplant computed tomography, and postoperative MRI to localize electrodes to parcellated brain regions and identify resection margins. We compared resected and spared band power abnormalities using the $D_{\mathrm{RS}}$ statistic and investigated its consistency over time (up to 9 days). A summary of the processing steps is shown in Figure 1.

### 2.1 Patients

We analyzed iEEG data from two cohorts: 39 patients with refractory focal epilepsy from the University College London Hospital (UCLH; Table 1), and 249 patients from the Restoring Active Memory (RAM) dataset. In the UCLH cohort, surgical outcomes were defined using the International League Against Epilepsy (ILAE) surgical outcome classification at 12 months postsurgery, with ILAE = 1 indicating seizure freedom.^16^ Our processing is broadly similar to previous work.^6^ We created a normative map of iEEG band power from the RAM iEEG data. The normative map was used as a baseline to compute the time‐varying iEEG band power abnormalities of the UCLH patients. Data were analyzed following approval from the Newcastle University Ethics Committee (2225/2017).

**TABLE 1:** Summary of University College London Hospital patient data.

Characteristic	ILAE = 1	ILAE>1	Test statistic
*n* (%)	16 (41%)	23 (59%)	
Age, mean (SD)	29.7 (4.3)	31.8 (9.5)	*p* = .41, *t* = −.84
Sex, M, F	7, 9	11, 12	*p* = .80, $\chi^{2}$ = .06
Temporal, extratemporal	8, 8	11, 12	*p* = .89, $\chi^{2}$ = .02
Side, left, right	10, 6	13, 10	*p* = .71, $\chi^{2}$ = .14
Contacts, *n*, mean (SD)	76.9 (27.5)	63.3 (22.8)	*p* = .1, *t* = 1.68
Recording duration, h, mean (SD)	122.9 (56.1)	123.1 (43.9)	*p* = .99, *t* = −.01

Abbreviations: F, female; ILAE, International League Against Epilepsy; M, male.

### 2.2 MRI processing

To generate a normative map, we localized RAM electrode coordinates to brain regions as described previously.^6^ In brief, we assigned electrodes to one of 128 regions from the Lausanne scale 60 atlas.^17^ We used FreeSurfer to generate volumetric parcellations of a Montreal Neurological Institute space template brain.^17^, ^18^ Each electrode contact was assigned to the closest gray matter volumetric region within 5 mm. If the closest gray matter region was >5 mm away, then the contact was excluded from further analysis. For UCLH data, a similar technique was used but applied in native space using the patient's own parcellated preoperative MRI.

To identify which regions were later resected in the UCLH cohort, we used previously described methods.^6^, ^19^ We registered postoperative MRI to the preoperative MRI and manually delineated the resection cavity. This manual delineation accounted for postoperative brain shift and sagging into the resection cavity. Electrode contacts within 5 mm of the resection were assigned as resected. Regions with >25% of their electrode contacts removed were considered as resected for downstream analysis.

### 2.3 iEEG processing

From each RAM patient, we extracted a single 30‐s segment of interictal iEEG data, recorded during a period of relaxed wakefulness. To approximate nonpathological brain dynamics, we excluded electrodes located in lesions or the seizure onset zone. We additionally removed visually and algorithmically identified noisy electrodes from the analysis. Each segment was then rereferenced to a common average reference, notch filtered at 60 Hz (2 Hz width, fourth order zero‐phase Butterworth filter), band pass filtered from .5 to 80 Hz (fourth order zero‐phase Butterworth filter), and downsampled to 200 Hz.

In the UCLH cohort, we first divided each patient's continuous iEEG data into 30‐s nonoverlapping, consecutive time windows. We referenced each window of data to a common average reference, with any noisy channels (with outlier amplitude ranges) excluded from the computed average. Each segment was then notch filtered at 50 Hz, band pass filtered from .5 to 80 Hz (fourth order zero‐phase Butterworth filter), and downsampled to 200 Hz. Time windows with missing data were omitted from the analysis.

We then computed the iEEG band power of both the RAM and UCLH iEEG data. We first computed the power spectral density (PSD) of each electrode contact in each 30‐s iEEG segment with 2‐s, nonoverlapping windows. From each PSD, we used Simpson's rule to compute band power in five frequency bands: delta (1–4 Hz), theta (4–8 Hz), alpha (8–13 Hz), beta (13–30 Hz), and gamma (30–47.5 Hz, 52.5–57.5 Hz, 62.5–77.5 Hz). We chose the gamma band limits to omit electrical noise frequencies. We log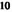 transformed the band power values and, for each iEEG segment and channel, normalized the set of five band power values to sum to 1, producing the relative log band power for each frequency band.

### 2.4 Creating iEEG band power normative map

To produce a normative map of relative log band power values, we averaged this measure across electrodes and patients within each region. First, for each RAM patient, we took the mean relative log band power across all of the patient's electrodes within each region. This step yielded patient‐specific relative log band power values at the region, rather than electrode, level. The normative map was then defined by the mean $\mu_{f,i}$ and SD $\sigma_{f,i}$ of the relative log band power in frequency band f and region i across the RAM patients. Regions with coverage from fewer than five subjects were excluded from the normative map and further analysis. Only one region (right accumbens) that was present in one UCLH patient was excluded from the downstream analysis due to lack of normative map coverage.

### 2.5 Computing time‐varying abnormalities and *D*_RS_

As with the RAM patients, we first computed relative log band power values at the region level for each UCLH patient within each 30‐s time window. As before, this transformation was achieved by taking the mean relative log band power across all of the patient's electrodes in region i. For each frequency band f, region i, and time window t, we then computed a z‐score $z_{f,i,t}$ by standardizing the patient's relative log band power $b_{f,i,t}$ by the normative map:

$$z_{f,i,t}=\frac{b_{f,i,t}-\mu_{f,i}}{\sigma_{f,i}}$$

We then defined the patient's band power abnormality for each region and time window as the maximum absolute *z*‐score across the five frequency bands, which captured any type of deviation from the region's normal band power. This approach has been previously successful in highlighting potentially abnormal epileptogenic tissue.^6^ Thus, each UCLH patient's iEEG recording was described by time‐varying abnormalities in their regions with electrode coverage.

To quantify the level of band power abnormalities in resected and spared regions, we computed the distinguishability of resected and spared tissue ($D_{\mathrm{RS}}$) for each time window in each UCLH patient. We defined $D_{\mathrm{RS}}$ as the area under the curve (AUC) for distinguishing resected and spared regions using band power abnormalities, with $D_{\mathrm{RS}}<$ .5 indicating higher abnormalities in resected regions and $D_{\mathrm{RS}}>$ .5 revealing higher abnormalities in spared regions.

### 2.6 Identifying interictal and peri‐ictal periods

For each UCLH patient, we labeled each time window as ictal, interictal, or peri‐ictal based on the patient's seizure times. From each patient's clinical annotations and reports, we obtained the times and durations of all recorded seizures, including subclinical seizures. The 30‐s time windows containing seizures were labeled ictal windows. Time windows within 1 h of an ictal time window, excluding the ictal time windows themselves, were labeled peri‐ictal windows. Finally, the remaining time windows were labeled interictal windows.

### 2.7 Identifying peaks and troughs of circadian cycles in a sleep marker

As a marker for sleep/wake periods, we identified each patient's circadian cycle in their alpha/delta band power ratio, averaged across all regions (see Figure S2 for details). Thirty‐second time windows with circadian cycle phases within π/4 radians of the cycle peaks and troughs were labeled as peak (indicative of wake) and trough (indicative of sleep) periods, respectively.

### 2.8 Code and data availability

Code and data to reproduce the main findings are available at : https://github.com/cnnp‐lab/2023Wang_Temporal‐stability.

## 3 RESULTS

We analyzed relative band power abnormalities in continuous iEEG data of 39 patients with focal epilepsy who underwent surgical resection. We focused on the presence of abnormalities in spared and resected brain regions in each patient, as captured by our measure $D_{\mathrm{RS}}$. In the following sections, we first present time‐varying abnormalities and $D_{\mathrm{RS}}$ in two sample patients. We then show the level of $D_{\mathrm{RS}}$ variability across patients and relate typical $D_{\mathrm{RS}}$ values to patient surgical outcomes. Finally, we compare $D_{\mathrm{RS}}$ values in interictal and peri‐ictal periods.

### 3.1 Location of abnormalities remains relatively stable

**FIGURE 2:**
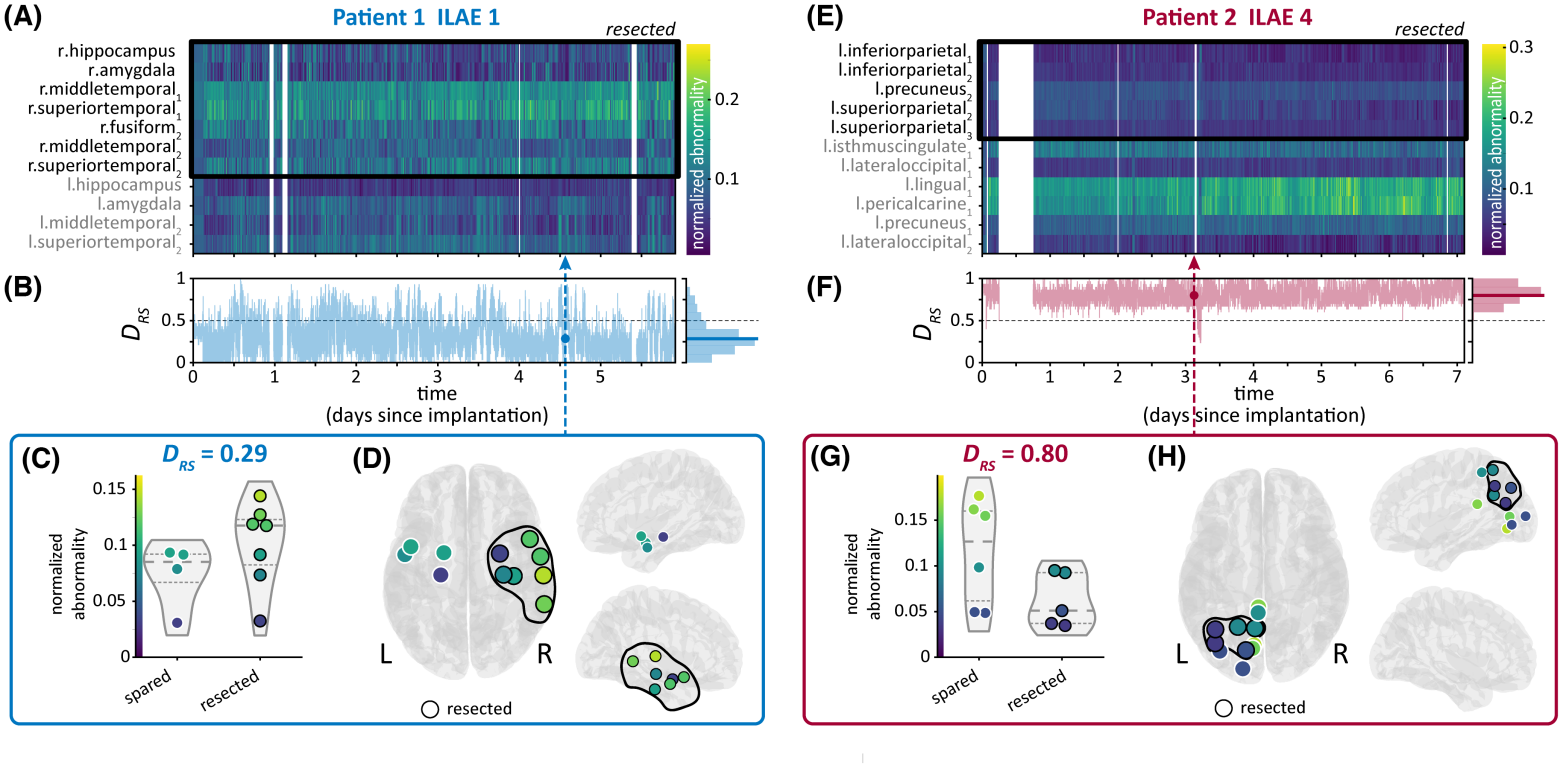
Time‐varying abnormalities and $D_{\mathrm{RS}}$ in sample Patients 1 (A–D) and 2 (E–H). (A, E) Heatmap of regional maximum absolute band power abnormalities, with each column corresponding to a 30‐s time window in the patient's intracranial electroencephalographic recording. Abnormalities in each time window are normalized to sum to 1, thus showing each region's contribution to the total abnormality in that time window. Resected regions are outlined with a black box. (B, F) Time‐varying $D_{\mathrm{RS}}$ computed from band power abnormalities. Histogram to the right of each plot shows the distribution of $D_{\mathrm{RS}}$ in each recording, with the median $D_{\mathrm{RS}}$ marked with a bold horizontal line. The circle and dashed vertical line mark a sample time window that had a $D_{\mathrm{RS}}$ equal to the patient's median $D_{\mathrm{RS}}$. $D_{\mathrm{RS}}=.5$ is also shown with a dashed black line for reference. (C, G) Normalized abnormalities of spared and resected regions in the sample time window with $D_{\mathrm{RS}}$ equal to the patient's median $D_{\mathrm{RS}}$. Quartiles of the abnormality distributions are marked with dashed lines. (D, H) The same abnormalities on a brain surface from top and side views. ILAE, International League Against Epilepsy.

Figure 2A shows the time‐varying abnormalities of an sample patient (Patient 1), who was seizure‐free following surgery (ILAE = 1). Although there was some spatial variability in abnormalities across the recording, abnormalities tended to be higher in resected brain regions. As such, $D_{\mathrm{RS}}$ was <.5 in most time windows, and the median $D_{\mathrm{RS}}$ across the recording was .29 (Figure 2B). Figure 2C,D, which show the abnormalities of a sample time window with $D_{\mathrm{RS}}=$ .29, further demonstrating the presence of higher abnormalities in resected brain regions in this patient.

Meanwhile, Patient 2 had a poor surgical outcome (ILAE = 4). As in Patient 1, regional abnormalities were relatively consistent across Patient 2's recording (Figure 2E). However, in Patient 2, higher abnormalities were located in spared regions, producing a $D_{\mathrm{RS}}$ > .5 across almost the entire recording and a high median $D_{\mathrm{RS}}$ of .8 (Figure 2F–H). Thus, in both patients, the median $D_{\mathrm{RS}}$ corresponds to postsurgical outcome and is relatively stable over time.

### 3.2 Median *D*_RS_ over time separates surgical outcomes

**FIGURE 3:**
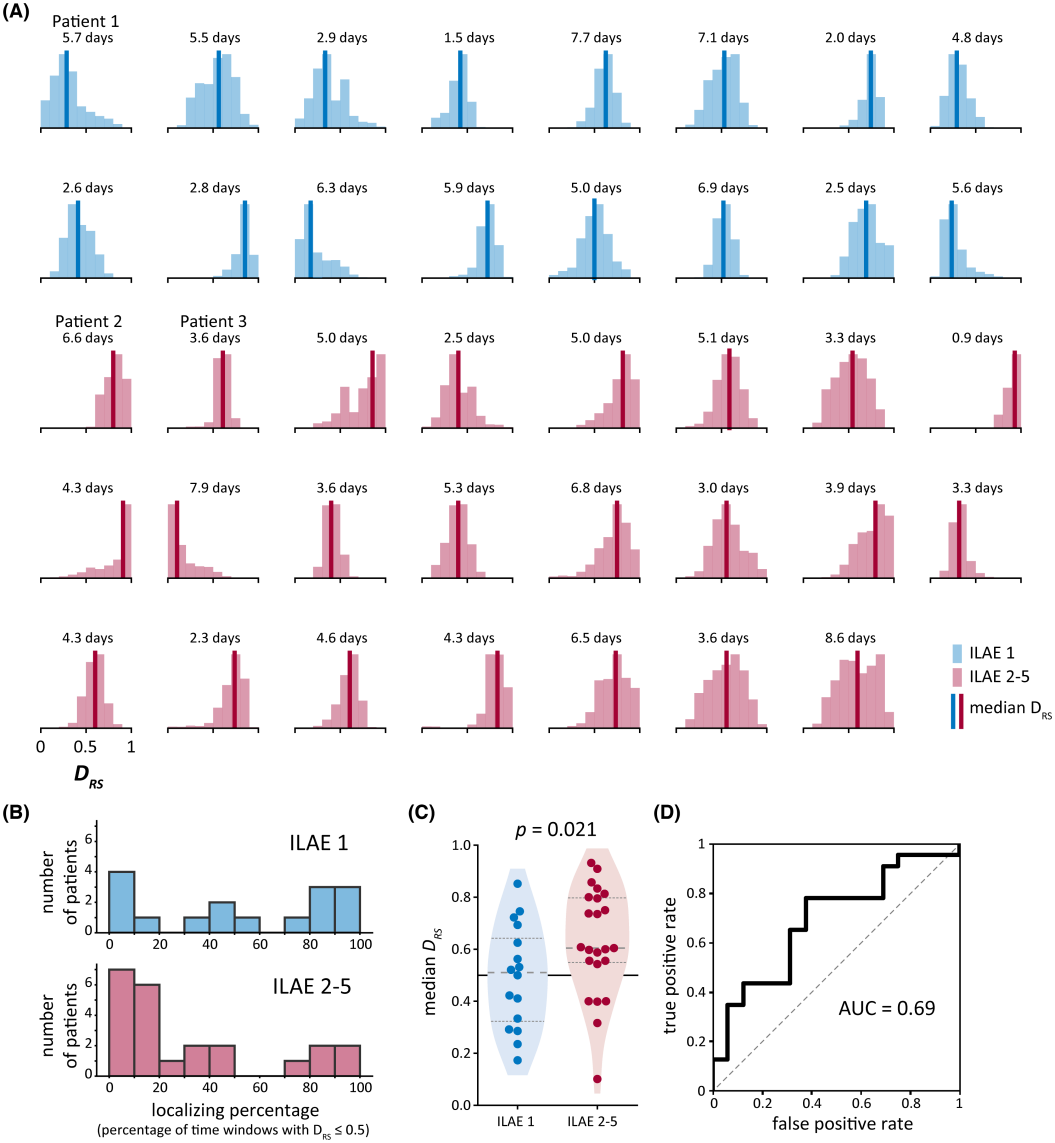
Variability in $D_{\mathrm{RS}}$ across patients. (A) Distribution of $D_{\mathrm{RS}}$ in each patient's intracranial electroencephalographic recording, as shown in sample patients in Figure 2B,F. Bold vertical lines show the median of each distribution. Number of days of data used to compute each distribution is also provided. (B) Localizing percentage of time windows (i.e., percentage of time windows with $D_{\mathrm{RS}}$ ≤ .5) in our cohort. (C) Comparison of median $D_{\mathrm{RS}}$ in patients who were seizure‐free (International League Against Epilepsy [ILAE] = 1) versus not seizure‐free (ILAE = 2–5) after surgery. Quartiles of the $D_{\mathrm{RS}}$ distributions are marked with dashed lines. (D) Receiver operator characteristic curve using median $D_{\mathrm{RS}}$ as a binary classifier of patient surgical outcome. AUC, area under the curve.

We next investigated variability in $D_{\mathrm{RS}}$ in all 39 patients in our cohort. Figure 3A shows the distribution of $D_{\mathrm{RS}}$ values in each patient's iEEG recording. Although there was within‐patient variability in $D_{\mathrm{RS}}$, most distributions were unimodal, with $D_{\mathrm{RS}}$ fluctuating around a particular value. Thus, although $D_{\mathrm{RS}}$ could differ between time windows, it was not random or highly variable.

As a simple measure of consistency in $D_{\mathrm{RS}}$, we computed the percentage of time windows in each patient with a $D_{\mathrm{RS}}$ value of ≤.5 (Figure 3B). We refer to this value as the “localizing percentage” of time windows; a high percentage indicates that abnormalities were consistently higher in the patient's resected brain regions, and thus localized to the hypothesized EZ. For example, in Patient 1 (Figure 2A–D), the localizing percentage was 87.2% due to consistently high abnormalities in resected regions. Low localizing percentages, which resulted from consistently high $D_{\mathrm{RS}}$ values, revealed that abnormalities were instead usually higher in spared regions, as in Patient 2 (Figure 2E–H; localizing percentage = .3%). In 28 of the 39 patients (72%), the localizing proportion was either low (≤20%) or high (≥80%). Thus, in most patients, $D_{\mathrm{RS}}$ values were either consistently > or <.5, indicating a consistent relationship between spared and resected abnormalities.

We then determined whether each patient's typical $D_{\mathrm{RS}}$ value, as captured by the median of their $D_{\mathrm{RS}}$ distribution, was associated with surgical outcome. Median $D_{\mathrm{RS}}$ was higher in patients who were not seizure‐free (ILAE = 2–5) versus seizure‐free (ILAE = 1) after surgical resection (p = .021, one‐sided Wilcoxon rank sum test; Figure 3C), and the AUC when using $D_{\mathrm{RS}}$ as a binary classifier of patient surgical outcome was .69 (Figure 3D). Patients who were not seizure‐free had median $D_{\mathrm{RS}}$ values of >.5 (p = .006, one‐sided Wilcoxon signed rank test), indicating higher abnormalities in spared brain regions in this group. However, seizure‐free patients did not have median $D_{\mathrm{RS}}$ values of <.5 (p = .455, one‐sided Wilcoxon signed rank test).

We also explored the amount of data needed to estimate patient $D_{\mathrm{RS}}$ for surgical outcome predictions (Figure S1). We found that estimating $D_{\mathrm{RS}}$ from even a small number (e.g., five) of randomly sampled, nonconsecutive 30‐s segments provided better and more consistent estimates of surgical outcome than using only one 30‐s segment per patient. No substantial improvement was found beyond 30 segments. In contrast, estimating $D_{\mathrm{RS}}$ from increasing numbers of consecutive segments barely improved outcome prediction.

### 3.3 Interictal and peri‐ictal time windows perform similarly at distinguishing patient surgical outcomes

**FIGURE 4:**
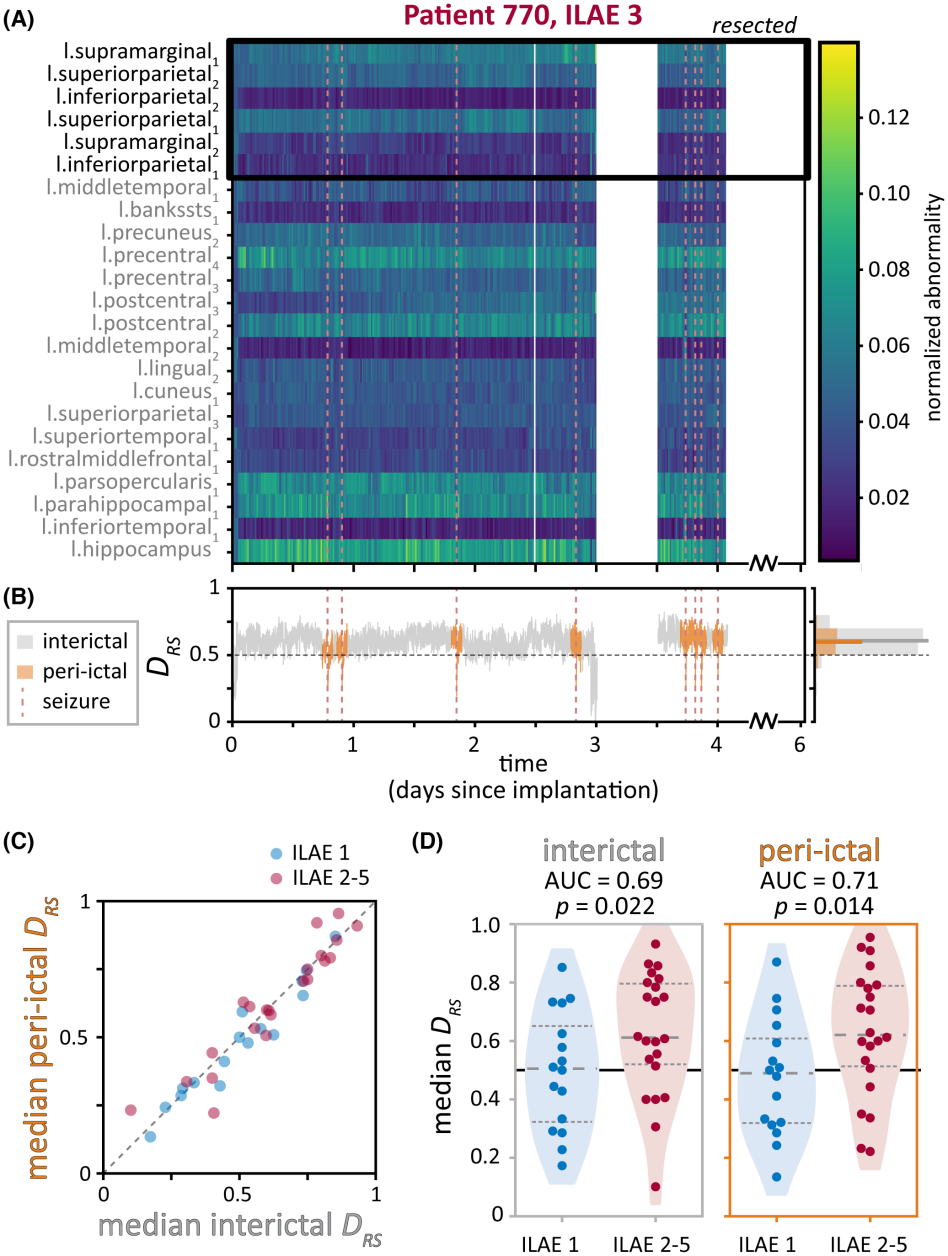
$D_{\mathrm{RS}}$ in interictal and peri‐ictal periods. (A, B) Interictal and peri‐ictal $D_{\mathrm{RS}}$ in sample Patient 3. Seizure times are marked with vertical dashed red lines. (A) Heatmap of time‐varying regional band power abnormalities (normalized to sum to 1). Resected regions are outlined with a black box. (B) Time‐varying $D_{\mathrm{RS}}$, colored by whether the time window was interictal (gray) or peri‐ictal (orange). Histogram to the right shows the distribution of $D_{\mathrm{RS}}$ and median value (bold horizontal lines) in each time period. (C, D) Interictal and peri‐ictal $D_{\mathrm{RS}}$ across patients. (C) Median peri‐ictal $D_{\mathrm{RS}}$ versus median interictal $D_{\mathrm{RS}}$ of each patient, colored by patient surgical outcome. (D) Comparison of median interictal (left) and peri‐ictal (right) $D_{\mathrm{RS}}$ in patients who were seizure‐free versus not seizure‐free after surgery. Quartiles of the $D_{\mathrm{RS}}$ distributions are marked with dashed lines. AUC, area under the curve; ILAE, International League Against Epilepsy.

Finally, we determined whether $D_{\mathrm{RS}}$ differed between interictal and peri‐ictal (defined as within 1 h of a seizure) periods within each patient. Figure 4A shows the time‐varying abnormalities of Patient 3, with seizure times marked with red dashed lines. The pattern of abnormalities appears relatively similar across the recording, regardless of the proximity to seizures. Likewise, Patient 3's $D_{\mathrm{RS}}$ was similar in interictal and peri‐ictal periods (Figure 4B), and the patient had almost the same median interictal and peri‐ictal $D_{\mathrm{RS}}$ (.61 and .60, respectively).

Across patients, we also observed that each patient had similar median interictal and median peri‐ictal $D_{\mathrm{RS}}$ (Figure 4C). The median values of each time period were not different either across all patients or within patients with the same surgical outcome (p = .17 for all patients, p = .09 for ILAE = 1 patients, p = .68 for ILAE = 2–5 patients, two‐sided Wilcoxon signed rank tests). As such, median interictal and median peri‐ictal $D_{\mathrm{RS}}$ also performed similarly at distinguishing patients by their surgical outcomes, with AUCs of .69 and .71, respectively (p = .022 and p = .014, one‐sided Wilcoxon rank sum tests; Figure 4D).

Similarly, we found no consistent influences of our sleep/wake marker on $D_{\mathrm{RS}}$ or surgical outcome predictions (Figure S2).

## 4 DISCUSSION

We have investigated the temporal stability of interictal band power abnormality patterns on iEEG relative to a normative map over timescales of multiple days. We found that in most patients, the localizing percentage of time windows was either low or high, indicating that abnormalities were consistently higher in either spared regions or resected regions, respectively, across each recording. We further demonstrated that the spatial distribution of abnormalities was temporally stable in terms of its localization ability, even peri‐ictally. Furthermore, we reproduced the previously reported separation of patients by postsurgical outcome based on median distinguishability of spared and resected tissue in each patient.

These findings have important implications for the practical application of interictal abnormality detection in presurgical evaluation with iEEG. Our results suggest that estimating a median abnormality map is sufficient to obtain localizing information. Additionally, we found that this estimate is best achieved by randomly sampling at least several short (e.g., 30 s) segments of iEEG data, rather than using a single larger window of continuous data. Interestingly, even peri‐ictal segments could be used to obtain an interictal abnormality map, although we still suggest avoiding segments with obvious ictal and peri‐ictal phenomena. Practically speaking, the suitability of peri‐ictal segments makes adopting the method immediately feasible, as it does not require additional manual screening or large amounts of continuous iEEG data handling. Beyond invasive iEEG recordings, our results hint at the possibility that noninvasive electrophysiological abnormality maps (e.g., based on scalp EEG, MEG) are likely also relatively stable over time. This hypothesis is supported by a recent MEG study mapping abnormalities across multiple epochs.^8^

Our findings are encouraging, especially when compared to other traditional interictal markers of epileptogenic tissue such as interictal spikes. Apart from the previously demonstrated added value of band power abnormality compared to interictal spikes in our cohort,^6^ our results here also seem to suggest that band power abnormality is temporally stable enough as a biomarker. It is unknown whether a similar stability is seen with interictal spikes, as spike load is reported to be generally higher during sleep,^20^ the pattern and location can vary depending on brain state,^1^ and a minimum analysis period of at least 24 h has been recommended.^1^ We encourage future work to evaluate and compare the temporal stability of more electrophysiological markers directly in the context of localization and validate it with surgical resection and outcome information.

Despite the temporal stability in terms of localization ability, our data also clearly show some level of temporal fluctuations in band power abnormalities. These fluctuations do not hamper the relative stability of distinguishability of resected and spared tissue. In particular, the circadian cycle in iEEG alpha/delta ratio, which we used as a marker of sleep/wake periods, did not consistently impact $D_{\mathrm{RS}}$ or surgical outcome predictions. Nevertheless, temporal fluctuations in abnormalities are present, and in some patients clearly structured in time (see, e.g., Figure 2A, region l.middletemporal_2_ for a circadian fluctuation in band power abnormality). These temporal fluctuations could reveal different pathological subnetworks and relate to seizure occurrence,^21^ for example, when they coalesce in space and time.^22^, ^23^, ^24^ It is also possible that they are related to seizure severity or other temporally modulated properties of seizures.^9^, ^25^, ^26^ The notion of fluctuating pathological subnetworks may also suggest alternative treatment strategies of network resections, disconnections, or closed‐loop neuromodulation of subnetworks.^22^, ^23^, ^24^ Finally, these abnormalities may not be epileptogenic per se, but relate to other temporally changing impairments in, for example, mood or cognitive performance. Future research should not neglect these time‐varying aspects simply due to our reported temporal stability in terms of localization performance, as these aspects may be the key to understanding fundamental mechanisms of epilepsy.

An important step for making band power abnormalities more specific and predictive is to account for the normative map of band power in various brain states. Sleep and vigilance states must be accounted for, as their band power changes are well known and described in healthy humans.^27^ Although we did not observe consistent sleep/wake differences in $D_{\mathrm{RS}}$, a sleep normative map may highlight sleep‐specific pathological dynamics by revealing deviations from normal sleep states. Cognitive and mood states have also been reported in terms of electrographic correlates.^28^, ^29^ We expect that by accounting for brain states and other potential confounds, the abnormalities will become more specific to epileptogenic tissue. In clinical practice, we envisage one or multiple short, well‐controlled abnormality mapping paradigms during iEEG monitoring with a straightforward but well‐defined state/task.

Another practical consideration for future work is how band power abnormality from iEEG information should be used. Here, we followed a previously used and straightforward method of measuring the distinguishability of resected and spared tissue ($D_{\mathrm{RS}}$) and related it to surgical outcome. However, other measures besides $D_{\mathrm{RS}}$ may need to be considered, especially given the abovementioned possibility of nonepileptogenic abnormalities and the patient‐specific spatial sampling in iEEG. Other possibilities include measures that only focus on the abnormality of the resected tissue or the proportion of resected abnormalities (see, e.g., Owen et al.^8^).

In a practical clinical workflow, it is also unlikely that median band power abnormality from iEEG would be considered as standalone information to guide surgical planning. We therefore envisage a quantitative multimodal approach to guide surgical planning, and there are several ways to achieve this goal. For example, abnormalities across modalities could be compared directly to determine whether they are concordant. Alternatively, multiple proposed surgeries could be simulated, and hypothetical $D_{\mathrm{RS}}$ values from multiple modalities could be combined quantitatively^30^ to decide on the best proposed resection. Finally, information from multiple modalities may also guide implantation of iEEG electrodes, allowing for more relevant spatial sampling and better subsequent surgical planning.^31^

In summary, using continuously recorded iEEG, we have demonstrated that band power abnormality maps are temporally stable in terms of their localizing information, despite brain state and seizure‐related changes throughout the recording. This finding is an important cornerstone in establishing the feasibility of band power abnormality mapping to aid localization for presurgical evaluation. We encourage investigating temporal patterns in band power for increased predictive power and mechanistic insights into epilepsy.

## FUNDING INFORMATION

P.N.T. and Y.W. are both supported by UKRI Future Leaders Fellowships (MR/T04294X/1, MR/V026569/1). M.P. and J.J.H. are supported by the Centre for Doctoral Training in Cloud Computing for Big Data (EP/L015358/1). J.S.D. and J.d.T. are supported by the NIHR UCLH/UCL Biomedical Research Centre. B.D. receives support from the NIH National Institute of Neurological Disorders and Stroke U01‐NS090407 (Center for SUDEP Research) and Epilepsy Research UK.

## CONFLICT OF INTEREST STATEMENT

None of the authors has any relevant conflict of interest to disclose.

## ETHICS STATEMENT

The study was approved by the NHNN and Institute of Neurology Joint Research Ethics Committee. We analysed the data following Newcastle University Ethics Committee approval (ref: 1804/2020). We confirm that we have read the Journal's position on issues involved in ethical publication and affirm that this report is consistent with those guidelines.

## Supporting information


FIGURE S1
FIGURE S2

## Data Availability

The data that support the findings of this study are available from the corresponding author upon reasonable request.
