# Supplementary material for: Temporal stability of intracranial electroencephalographic abnormality maps for localizing epileptogenic tissue
Source: Epilepsia. 2023 Jun 6;64(8):2070–80. doi: 10.1111/epi.17663 (PMC10962550; doi:10.1111/epi.17663)
Supplement: Supplementary file 1 — FIGURE S1 FIGURE S2 [file EPI-64-2070-s001.docx]

# Supplementary

# Median $\boldsymbol{D}_{\boldsymbol{RS}}$ of randomly sampled non-consecutive segments distinguishes patients by surgical outcome

In figure 3, we showed that the median $D_{RS}$ of each patient’s recording distinguishes patients by their surgical outcomes (ILAE 1 versus ILAE 2-5). In this supplementary analysis, we investigated the number of 30 s segments needed to estimate patient $D_{RS}$ for distinguishing patient outcomes. In each patient, we randomly sampled $n$ non-consecutive and $n$ consecutive segments, with $n$ = 1, 5, 10, 30, 60, 90, and 120. Segments with missing data were excluded from the sampling procedure. Note that for the non-consecutive sampling case, temporally neighboring segments could be selected by chance, but in most cases the segments will be temporally distributed throughout the patient’s recording.

For each scenario (i.e., sampling type and $n$), we sampled $n$ segments from each patient 10,000 times and then computed the median $D_{RS}$ of each patient’s sampled segments. Patient median $D_{RS}$ values were then used as a binary classifier of patient surgical outcomes, as in Fig. [3](#across_patients)C-D.

Fig. [S1](#s_sampling_fig) shows how surgical outcome AUCs change for non-consecutive and consecutive sampling as the number of segments increases. For non-consecutive sampling, as the number of segments increases, the mean AUC increases and AUC variability decreases. Even a relatively small number of segments ($n$ = 5) has a marked effect on these features, with the impact of $n$ diminishing above $n$ = 30. Meanwhile, increasing sample size when using consecutive sampling barely changes AUC estimates. Note that increasing number of consecutive samples is comparable to increasing the duration of the iEEG segments.


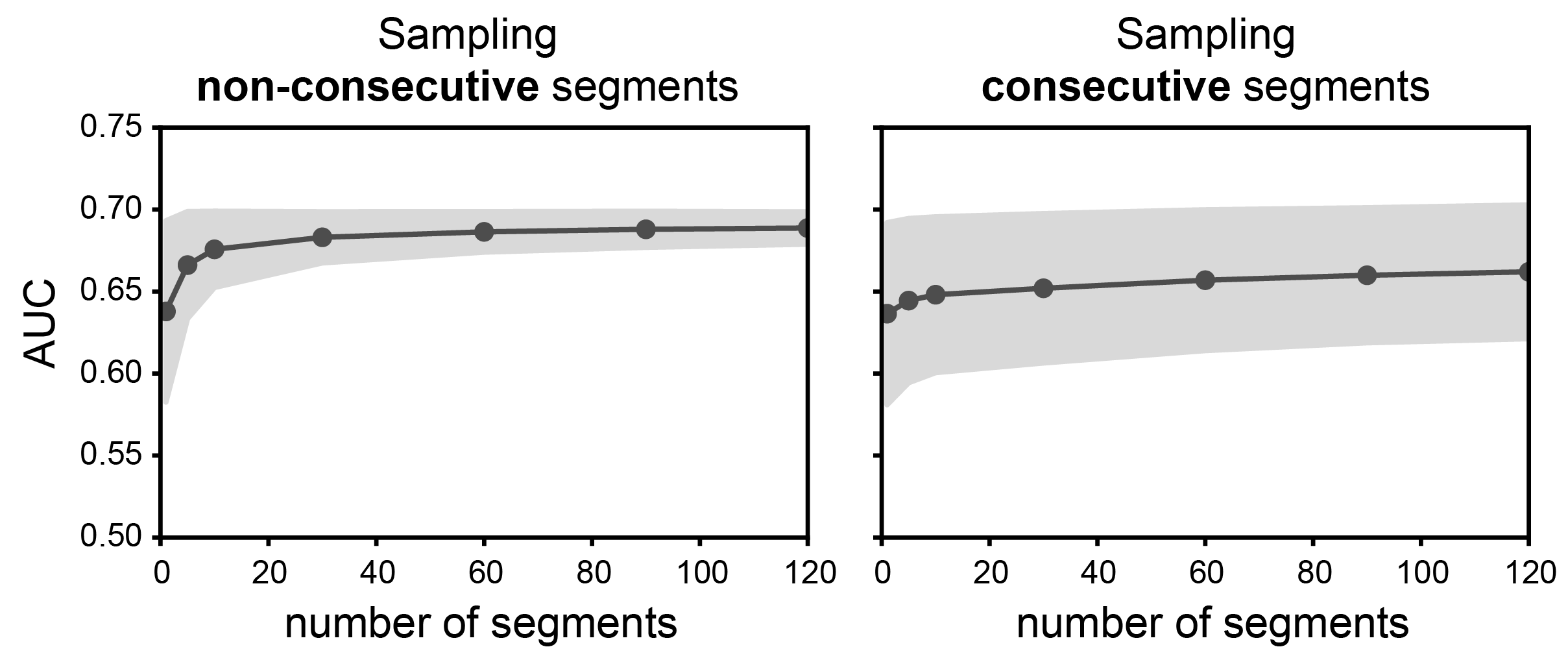


**Figure S1: Distinguishing surgical outcomes using** $D_{RS}$ **estimates from random samples of patient recordings.** AUC when using median $D_{RS}$, estimated from randomly sampled non-consecutive (left) or consecutive (right) 30 s iEEG segments, as a binary classifier of patient surgical outcomes. Each plot shows the mean AUC $\pm$ one standard deviation versus the number of sampled segments.

# $\boldsymbol{D}_{\boldsymbol{RS}}$ and outcome predictions do not vary over circadian cycles in iEEG alpha/delta ratio

We investigated whether $D_{RS}$ varies over established circadian cycles in iEEG dynamics. Specifically, the alpha/delta band power ratio can serve as an approximation of sleep/wake periods due to the presence of higher alpha power in wake periods and higher theta power in sleep. As such, this marker has previously been used to explore the impact of sleep/wake on other localization biomarkers, including spikes^1^.

We first extracted peak (indicative of wake) and trough (indicative of sleep) periods in the circadian cycle of the time-varying alpha/delta ratio of each patient (Fig. [S2](#s_circ_fig)A). For each 30 s segment of channel band power, we transformed alpha and theta band power values $b$ by computing $log10\left( b+1 \right)$, then computed the mean transformed band power values in each region. The alpha/delta ratio at the region level was then averaged across regions to yield a single time-varying alpha/delta ratio for each recording. The ratio time series was filtered from 2/3 to 4/3 cycles/day (6th order zero-phase Butterworth bandpass filter) to extract circadian fluctuations, and phases of the circadian cycle were computed using the Hilbert transform. Cycle peaks and troughs were defined as segments within $\pi/4$ radians of the peak ($0$ radians) and trough ($\pi$ radians) phases, respectively. These segments are highlighted in orange and purple, respectively, in Fig. [S2](#s_circ_fig)A.

We then compared the $D_{RS}$ and surgical outcome predictions based on peak and trough periods. We excluded two patients with fewer than six total hours of either peak or trough periods from this analysis. For the remaining 37 patients, we computed the median $D_{RS}$ of their peak and trough periods. Median alpha/delta peak $D_{RS}$ was not significantly different from median alpha/delta trough $D_{RS}$ at a cohort level ($p$ = 0.094 , two-sided Wilcoxon signed rank tests) or in patients who were seizure free or not seizure free after surgery ($p$ = 0.158 and $p$ = 0.173, respectively, two-sided Wilcoxon signed rank tests) (Fig. [S2](#s_circ_fig)B). The median $D_{RS}$ of both alpha/delta peak and trough periods performed similarly at distinguishing patients by surgical outcomes (AUC = 0.70 and $p$ = 0.021 for peak, AUC = 0.69 and $p$ = 0.025 for trough, one-sided Wilcoxon rank sum tests) (Fig. [S2](#s_circ_fig)C).


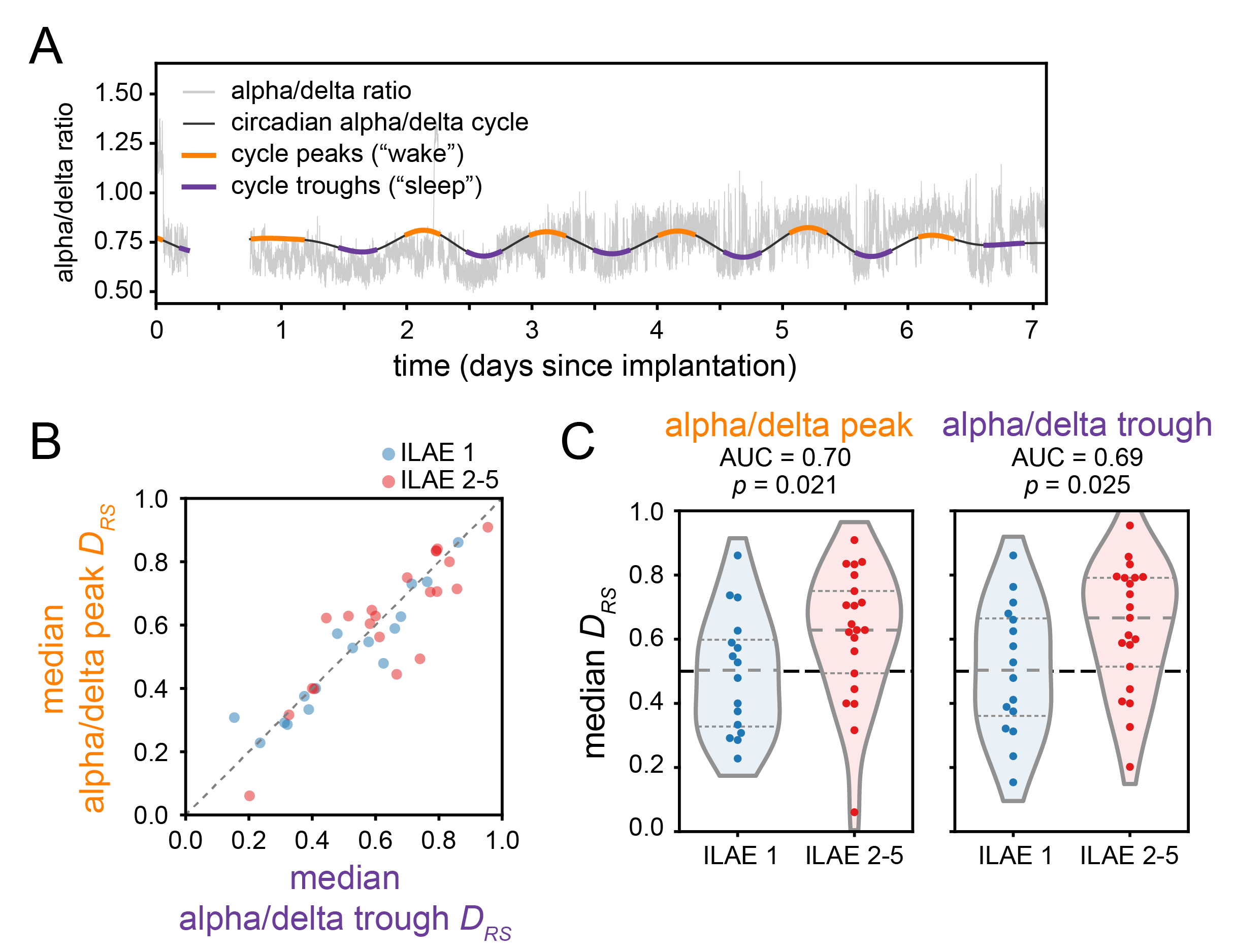


**Figure S2:** $D_{RS}$ **in periods of with low and high alpha/delta ratio during the circadian alpha/delta cycle.** A) Time-varying alpha/delta ratio in an example patient’s recording, with the peaks and troughs of the circadian cycle highlighted in orange and purple, respectively. B) Median $D_{RS}$ of alpha/delta peak periods versus median $D_{RS}$ of alpha/delta trough periods across patients, colored by patient surgical outcome. C) Comparison of median $D_{RS}$ of alpha/delta peak (left) and alpha/delta trough (right) periods in patients who were seizure free versus not seizure free after surgery. Quartiles of the $D_{RS}$ distributions are marked with dashed lines.
